# Early Neural Cell Death Is an Extensive, Dynamic Process in the Embryonic Chick and Mouse Retina

**DOI:** 10.1155/2013/627240

**Published:** 2013-04-09

**Authors:** Teresa Chavarría, Jimena Baleriola, Raquel Mayordomo, Flora de Pablo, Enrique J. de la Rosa

**Affiliations:** ^1^3D Lab (Development, Differentiation, Degeneration), Department of Cellular and Molecular Medicine, Centro de Investigaciones Biológicas, CSIC, C/Ramiro de Maeztu 9, E-28040 Madrid, Spain; ^2^Department of Anatomy, Cellular Biology and Zoology, Centro Universitario de Plasencia, Universidad de Extremadura, Av. Virgen del Puerto, E-10600 Plasencia, Spain; ^3^Instituto de Salud Carlos III, C/Sinesio Delgado 4, E-28029 Madrid, Spain

## Abstract

Orchestrated proliferation, differentiation, and cell death contribute to the generation of the complex cytoarchitecture of the central nervous system, including that of the neuroretina. However, few studies have comprehensively compared the spatiotemporal patterns of these 3 processes, or their relative magnitudes. We performed a parallel study in embryonic chick and mouse retinas, focusing on the period during which the first neurons, the retinal ganglion cells (RGCs), are generated. The combination of *in vivo* BrdU incorporation, immunolabeling of retinal whole mounts for BrdU and for the neuronal markers Islet1/2 and **β**III-tubulin, and TUNEL allowed for precise cell scoring and determination the spatiotemporal patterns of cell proliferation, differentiation, and death. As predicted, proliferation preceded differentiation. Cell death and differentiation overlapped to a considerable extent, although the magnitude of cell death exceeded that of neuronal differentiation. Precise quantification of the population of recently born RGCs, identified by BrdU and **β**III-tubulin double labeling, combined with cell death inhibition using a pan-caspase inhibitor, revealed that apoptosis decreased this population by half shortly after birth. Taken together, our findings provide important insight into the relevance of cell death in neurogenesis.

## 1. Introduction

The complex cellular arrangement of the vertebrate central nervous system, including the neuroretina, largely arises from the apparently undifferentiated, pseudostratified neuroepithelium. During early embryonic development, neuroepithelial cells undergo sequential proliferation and differentiation to give rise to neurons and glial cells. In addition, proliferating and recently differentiated neural cells are subjected to early neural cell death. Consequently, the neuroepithelium changes progressively and rapidly: the proportion of proliferating cells decreases as development proceeds, while the number of differentiated cells increases. Dead cells are rapidly removed from the tissue by neighboring cells or microglia. As these dynamic changes overlap, it is difficult to determine the actual impact of cell death on proliferation or differentiation. Moreover, the physiological role of early neural cell death in neurogenesis remains unclear [1–5].

The neuroretina is a highly useful model system [6–9], and our understanding of cell death has been significantly advanced by studies of retinal development (reviewed in [10]). The vast majority of cells present in the neuroretina in early developmental stages are generated from the optic cup neuroepithelium. Unlike other parts of the central nervous system, nontangential migration does not occur, and few microglial or endothelial cells are observed at these early stages [11]. 

In the present study, we focused on the period when the first neurons, the retinal ganglion cells (RGCs), are generated from proliferating neuroepithelial cells, in order to characterize the relative spatial distribution and magnitude of proliferation, differentiation, and cell death in embryonic chick and mouse retinas. In addition, using the window-labeling technique [12], we demonstrated that recently generated RGCs die in significant numbers in the embryonic chick retina. An accurate assessment of the impact of early neural cell death in comparable developmental stages in chick and mouse retinas may facilitate the design of future experiments to further our understanding of its functional role in neurogenesis.

## 2. Materials and Methods

### 2.1. Chick and Mouse Embryos

Fertilized White Leghorn eggs were incubated at 38.4°C in a humidified incubator for the desired period. Embryonic age was described in HH stages [13]. C57BL/6J mice were reared in local facilities at 20°C on a 12-hour light/dark cycle. Mice were crossed and the morning on which the vaginal plug was detected was designated embryonic day (E) 0.5. Mouse embryos were collected from pregnant females. Animals were handled and euthanized in accordance with the European Union guidelines for animal research and the ARVO guidelines for the use of animals in ophthalmic and vision research, and all protocols were approved by the local bioethics committee.

### 2.2. *In Vivo* BrdU Incorporation

Cell proliferation was determined by the incorporation of the thymidine analogue 5-bromo-2′-deoxyuridine (BrdU; Sigma, St. Louis, MI, USA) to DNA during the S-phase of the cell cycle. BrdU-labeled cells were visualized by specific immunolabeling (see below). 

For chick retina labeling, BrdU was administered *in ovo* via a small opening in the egg shell and a small incision in the embryonic membrane. BrdU (2.5 *μ*g/egg) was deposited using a micropipette in a final volume of 25 *μ*L of PBS. Sellotape was used to seal the shell opening, and the eggs were incubated for a further 10 h before processing [14]. 

For window labeling [12], BrdU incorporation was stopped after 1 hour by the addition of an excess of thymidine (1.25 mg/egg) in a final volume of 16.7 *μ*L, injected as described above. The egg was then sealed again and incubated for the indicated period. 

Where indicated, chick embryos were also treated *in ovo *with Boc-D-fmk (Calbiochem, San Diego, CA, USA), a pan-caspase inhibitor that largely prevents caspase-dependent programmed cell death [14]. In this case, in parallel with the thymidine administration, 5 *μ*L of 38 *μ*M Boc-D-fmk were administered around the optic area of the chick embryo. 

Mouse embryos were labeled by intraperitoneal BrdU injection (10 mg/Kg) in pregnant females, and the embryo retinas were processed 10 h later.

In all cases, the animals were euthanized and staged, the eyes were enucleated, and the retinas were dissected, whole-mounted on nitrocellulose and fixed overnight in 4% paraformaldehyde (w/v) in 0.1 M phosphate buffer (pH 7.4). In previous studies, and occasionally in the present study, retinal cryosections were used to confirm the labeling of all retinal layers, as well as the spatial allocation of the labeled cells.

### 2.3. Immunostaining

Proliferating cells and young neurons were visualized in retinal whole mounts by specific immunostaining [14]. *β*III-tubulin is a specific constituent of the neuronal microtubules. Islet1/2 is a neuronal transcription factor. At the early stages studied, both are specific for RGCs.

Fixed retinal whole mounts were first permeated for 2 h with 1% (w/v) Triton X-100 in PBS and then treated with collagenase (20 U/mL, Sigma) for 20 min at 37°C. The retinas were incubated overnight at 4°C with primary antibodies against BrdU (1/1000; the anti-BrdU antibody G3G4 was generated by S. J. Kaufman and was obtained from the Developmental Studies Hybridoma Bank, maintained by the University of Iowa, Department of Biological Sciences, Iowa City, IA, USA), *β*III-tubulin (1/1000; polyclonal anti-rabbit Tuj-1, Covance, Paris, France), and Islet1/2 (1/200; the anti-Islet1/2 antibody 39.4D5 was generated by T. M. Jessell and was obtained from the Developmental Studies Hybridoma Bank). The retinas were then washed and incubated for 1 hour at RT with the corresponding AlexaFluor488-conjugated goat anti-mouse, AlexaFluor647-conjugated goat anti-mouse, AlexaFluor568-conjugated goat anti-rabbit, or AlexaFluor647-conjugated goat anti-rabbit secondary antibodies (all 1/200; Molecular Probes, Invitrogen, Carlsbad, CA, USA). For Islet1/2 staining in chick retinas, a biotin-conjugated goat anti-mouse secondary antibody was used. After washing, the retinas were incubated for 1 hour at RT with AlexaFluor647-conjugated streptavidin. PBS containing 2 mg/mL BSA, 100 mM glycine, and 0.25% (w/v) Triton X-100 was used for all dilutions and for washings between incubations. The retinas were mounted with DABCO 4% (w/v) (Sigma) and glycerol 70% (v/v) for confocal microscopy analysis.

For epitope retrieval, retinas were treated prior to incubation with the primary antibodies with 2 N HCl (for BrdU staining), or 10 mM sodium citrate (pH 6.0) and microwaves (for Islet1/2 staining).

### 2.4. Detection of Apoptosis

DNA fragmentation characteristic of apoptosis was visualized by TUNEL in retinal whole-mounts [14] using FITC-dUTP, following the manufacturer's instructions (Apoptosis Detection System, Promega, Madison, WI, USA). 

TUNEL staining was performed as described above for immunostaining but with an additional 10-minute incubation at 37°C with proteinase K (20 *μ*g/mL, Promega) and refixation prior to the TdT reaction. 

### 2.5. Isodensity Maps of Proliferating, Differentiated, and Apoptotic Cells

Isodensity maps define areas that share similar cell densities, which are represented using a colorimetric scale; warm colors correspond to high-cell densities and cold colors to low-cell densities. These maps allowed for comparison of the spatiotemporal progression of the different processes during early retinal development in chick and mouse embryos. 

After performing BrdU and Islet1/2 immunostaining, as well as TUNEL, retinal whole mounts were visualized using either a conventional or a confocal microscope. Positive cells were scored using a grid map.

### 2.6. Statistical Analysis

Student-Newman-Keuls post hoc tests were used for statistical analysis of the data. Each experimental point represents the mean ± standard deviation of at least 3 retinas from 3 different embryos (*P < 0.005).

## 3. Results

**Figure 1:**
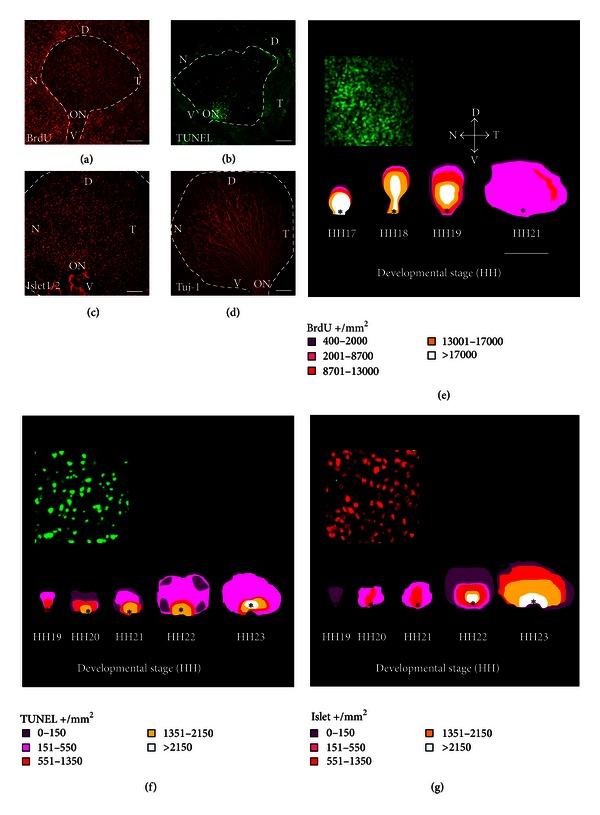
Overlapping cell processes in the embryonic chick retina. Representative labeling of cell proliferation (a), death (b), and differentiation (c), (d) in retinal whole mounts from chick embryos ((a), HH19; (b), HH20; (c), HH23; (d), HH21). Positive cells were scored in 4–6 retinas from different embryos and represented on isodensity maps of the different processes (e)–(g). The insets show positive cells at higher magnification, as employed for scoring. The main morphological features are labeled as follows. ON and *: optic nerve; D: dorsal; V: ventral; T: temporal; and, N: nasal. Scale bars represent 80 *μ*m (a)–(d), 500 *μ*m (e)–(g), and 40 *μ*m (insets).

**Figure 2:**
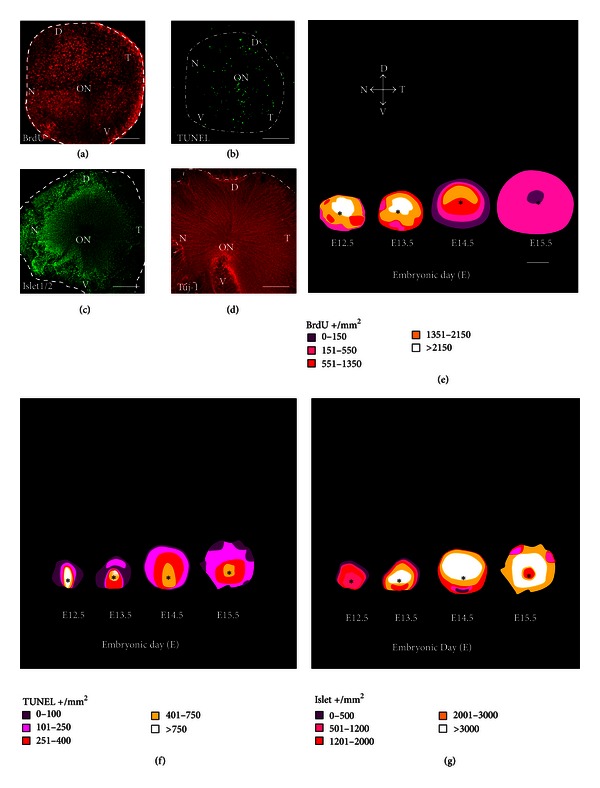
Overlapping cell processes in the embryonic mouse retina. Representative labeling of cell proliferation (a), death (b), and differentiation (c), (d) in retinal whole mounts from E13.5 mouse embryos. Positive cells were scored in 3–10 retinas from different embryos and represented on isodensity maps of the different processes (e)–(g). The main morphological features are labeled as follows: ON and *: optic nerve; D: dorsal; V: ventral; T: temporal; N: nasal. Scale bars represent 150 *μ*m (a)–(d) and 300 *μ*m (e)–(g).

**Figure 3:**
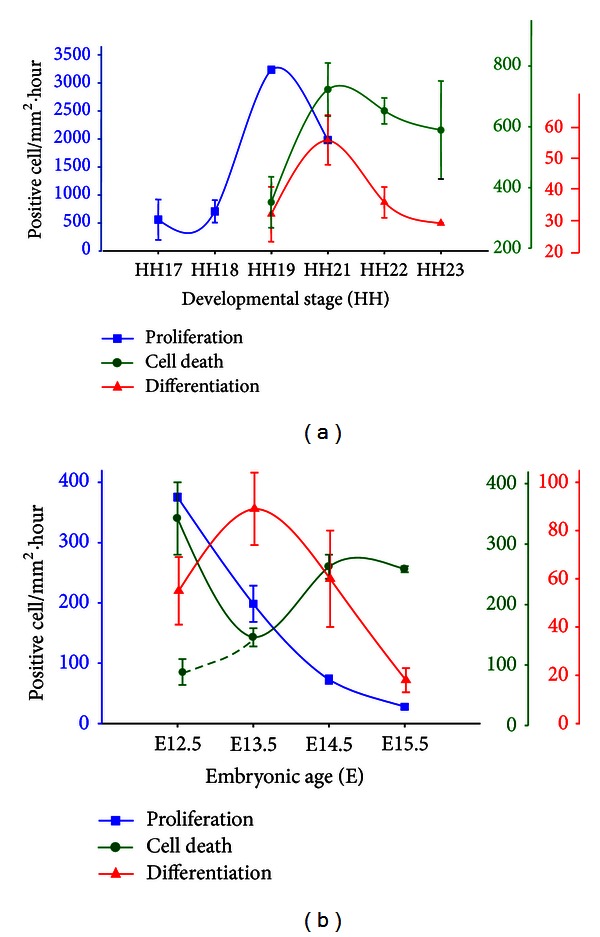
Relative magnitudes of cell proliferation, differentiation, and death in embryonic chick and mouse retinas. The results of the experiments described in Figures 1 and 2 were normalized per retinal surface area and hour, as explained in Section 3.2. Graphs represent the relative magnitude of cell proliferation (blue lines: BrdU-positive cells), differentiation (red lines: Islet1/2-positive cells), and death (green lines: TUNEL-positive cells) in chick (a) and mouse (b) embryonic retinas. The alternative slashed line representing cell death in the mouse retina indicates the magnitude of this process after subtracting the TUNEL-positive nuclei associated to the optic fissure, which plays a role in morphogenesis.

**Figure 4:**
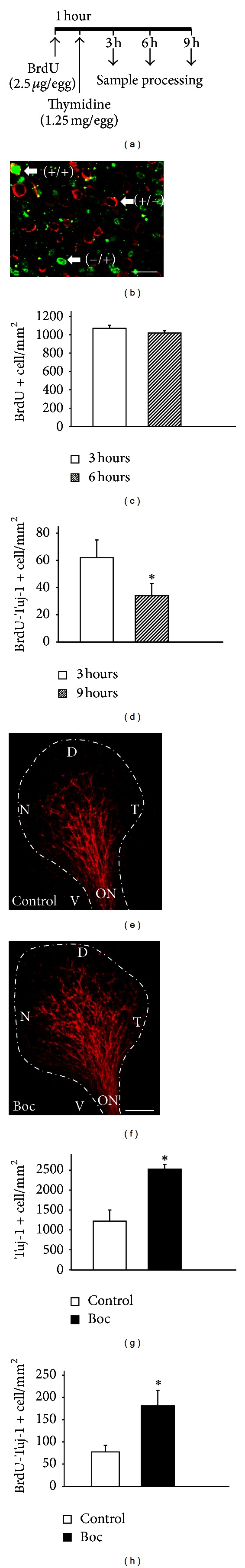
Effect of cell death on recently generated retinal ganglion cells. In HH21 chick embryos, *in ovo* window labeling (a) was used to visualize a defined population of recently generated neurons ((b); +/+; Tuj-1-positive in red; BrdU-positive in green). Proliferating cells ((b); −/+; BrdU-positive in green) were scored at different time points (c) to confirm complete closure of the window. Preexisting neurons were also visualized ((b); +/−; Tuj-1-positive in red). Recently generated neurons were scored at different time points (d). Retinal whole mounts treated with Boc-D-fmk exhibited an expanded neuronal domain as compared with untreated retinas, as visualized by Tuj-1 immunostaining (e) and (f). Total neurons (g) and recently generated neurons (h) were scored in control and Boc-D-fmk-treated embryos. The main morphological features are labeled as follows: ON: optic nerve; D: dorsal; V: ventral; T: temporal; N: nasal. Scale bars represent 20 *μ*m (b) and 80 *μ*m (e), (f). Graphs represent the mean ± SD of at least 3 retinas from different embryos. *P < 0.005.

In the present study, we analyzed comparable early embryonic stages in chicks (HH17 to HH23, approximately E2.5 to E4) and mice (E12.5 to E15.5) to determine the rate and spatial distribution of the main cellular processes that accompany retinal neurogenesis, namely, proliferation, differentiation, and cell death. During these periods, the first retinal neurons, the RGCs, are generated. We selectively labeled proliferating (BrdU-positive), differentiated (Islet1/2- and Tuj-1-positive), and dying (TUNEL-positive) cells in the neuroretina (Figures 1(a)–1(d) and Figures 2(a)–2(d)). Based on these labelings, we generated whole retina isodensity maps (Figures 1(e–g) and Figures 2(e–g); insets in Figures 1(e–g) show, at higher magnification, the respective scoring fields) and compared the relative magnitude of the different cellular processes (Figure 3). A second aim of the study was to accurately quantify the impact of early neural cell death on the population of recently born RGCs. This was achieved by scoring of cells that were double-labeled for BrdU and Tuj-1 using the window-labeling technique (Figures 4(a)–4(d)), and by inhibition of apoptosis with Boc-D-fmk, a pan-caspase inhibitor (Figures 4(e)–4(h)). These experiments were performed in the chick embryo, which is more amenable to this approach than the mouse embryo.

### 3.1. Proliferation, Differentiation, and Cell Death Coincide during Early Retinal Development and Exhibit Distinct but Overlapping Distribution Patterns

#### 3.1.1. Isodensity Maps: Cell Proliferation

Using the 10-hour *in vivo* BrdU incorporation protocol, we visualized the overall central-to-peripheral gradient of cell proliferation in both chick (Figures 1(a) and 1(e)) and mouse (Figures 2(a) and 2(e)) embryos, at all stages studied. At high magnification, this method permitted precise scoring (see inset in Figure 1(e)). The total numbers obtained were projected onto isodensity maps (Figures 1(e) and 2(e)), in which cell densities are represented on a colorimetric scale. In the chick neuroretina, we observed a marked decrease in the density of labeled cells at later stages, as expected during the transition from proliferating neuroepithelial cells to postmitotic neurons. A similar trend was observed in the mouse retina, although labeling was irregular in the most central area surrounding the optic nerve head.

#### 3.1.2. Isodensity Maps: Cell Death

TUNEL in retinal whole mounts demonstrated the overall central-to-peripheral gradient of retinal development (Figures 1(b), 1(f), 2(b), and 2(f)). Most if not all cells dying at specific stages of development were visualized using this approach (inset in Figure 1(f)), and their spatial distribution revealed more irregularities than observed for proliferating cells, likely reflecting the different phases of cell death (reviewed in [10]). Cell death associated with closure of the optic fissure, which plays a morphogenic role, was observed at early stages in the mouse retina (Figures 2(b) and 2(f)).

#### 3.1.3. Isodensity Maps: Cell Differentiation

An overall view of neuronal generation and differentiation was obtained by immunostaining for Islet1/2, a neuronal specific transcription factor (Figures 1(c) and 2(c)), and for *β*III-tubulin, a specific component of the neuronal cytoskeleton (Figures 1(d) and 2(d)). Islet1/2 immunostaining is restricted to the neuronal nuclei and allowed scoring of the neurons at each stage (inset in Figure 1(g)). As development proceeded, neurons accumulated following a central-to-peripheral gradient (Figures 1(g) and 2(g)). Central disruption of this pattern was observed in the E15.5 mouse retina, as also observed for proliferation and cell death.

### 3.2. Relative Magnitudes of Cell Proliferation, Differentiation and Death during Early Retinal Development

Although the isodensity maps provided valuable spatial information, precise comparison of the magnitude of each process was hindered by several peculiarities specific to each process. Neuroepithelial cells continuously progress through the cell cycle phases. The duration of the cell cycle in the early retinal neuroepithelium is approximately 12 hours ([15], and our own unpublished results in the chick retina). Accordingly, 10 hours of BrdU labeling should reflect a single transition through the S-phase of most mitotic cells. Dead cells are rapidly engulfed and degraded by neighboring cells and phagocytes in an estimated time ranging from 45 min to 2 hours [16, 17]. By contrast, postmitotic neurons accumulate over time. To compare the magnitude of the 3 processes in terms of actual cell numbers (Figure 3), we standardized cell numbers over a 1-hour interval. Raw numbers of labeled cells were first normalized to the area of the retina. TUNEL-positive nuclei were represented as scored. Numbers of BrdU-positive nuclei were divided by the 10-hour labeling period. The increase per hour in the number of Islet1/2-positive nuclei was calculated by subtracting the total number scored at 2 sequential stages and dividing the figure by the developmental interval, in hours, between the two.

As expected, proliferation was the most prevalent process in the retina in the early stages studied, particularly in the chick (Figure 3(a)), peaking at HH19, with over 3,000 labeled cells/mm^2^·hour. Proliferation was less prominent at stages earlier than HH19, and subsequently decreased, coinciding with a parallel increase in both differentiation and cell death, which suggests a correlation between the latter 2 processes. Strikingly, after normalization, the number of dead cells for the studied interval was 10- to 20-fold higher than that of differentiated cells.

In the embryonic mouse retina (Figure 3(b)), we observed a stepwise decrease in proliferation, beginning at 400 labeled cells/mm^2^·hour. Proliferation is likely higher prior to E12.5, however, these early stages are not well suited to retinal whole-mount processing. Cell death increased after differentiation, with the exception of the optic fissure, where it plays a morphogenic role. Cell death presented, with respect to differentiation, a greater delay than that observed in the chick, likely reflecting the slower rate of development in the mouse. As found in the chick retina, the magnitude of cell death at its peak was 3-fold greater than that of differentiation.

### 3.3. Recently Generated Retinal Ganglion Cells Are Subjected to Extensive Cell Death

The relative dynamics of differentiation and cell death (Figure 3) suggest that the latter preferentially affects recently generated neurons, particularly RGCs, during the developmental stages analyzed. To investigate this hypothesis and to more accurately determine the proportion of RGCs affected by cell death, we used the *in ovo* window-labeling technique (Figure 4(a)) [12] in HH21 chick embryos, the time point at which both differentiation and cell death peaked in our experimental conditions. A limited proportion of cycling neuroepithelial cells were labeled with BrdU (i.e., only those proceeding through the S-phase during a 1-hour interval), as incorporation was stopped after 1 hour by the addition of a 500-fold excess of nonlabeled thymidine. The fate and number of the labeled cells were determined by double immunolabeling for BrdU and Tuj-1 (Figure 4(b)), which visualized recently generated RGCs. While BrdU labeling was stable over time (Figure 4(c)), indicating complete closure of the labeling window, the number of BrdU/Tuj-1 double-labeled cells was reduced by 50%, revealing a dramatic impact of cell death on this specific neuronal population. Moreover, the addition of Boc-D-fmk, a pan-caspase inhibitor that interferes with early neural cell death [14], increased the total number of RGCs and restored the population of recently generated RGCs (Figures 4(e)–4(h)). 

## 4. Discussion

In our previous studies of the developing retina in chicks and mice (reviewed in [10]), we demonstrated the existence of an early phase of programmed cell death and characterized its regulation by different factors and signaling pathways. In the present study, we sought to characterize the magnitude of this process, as compared with other parallel processes that contribute to retinal development.

The analysis of retinal whole mounts was essential to characterize the occurrence of early neural cell death in the embryonic chick retina, as this process is dynamic and apoptotic cells are rapidly engulfed by neighboring cells and phagocytes. We extended this method to also analyze proliferation and differentiation, given the proposed coordinated regulation of all 3 processes in cell cycle exit and neurogenesis during neural development [18]. The resulting isodensity maps supported previous reports of a central-to-peripheral gradient of retinal development [19] and confirmed the spatiotemporal overlap of cell proliferation, differentiation, and death. 

Whole-mount analysis also allowed for a more precise quantification of the total number of cells undergoing each process. Actual cell numbers were normalized to retinal surface area and time, for ease of interpretation (Figure 3). Cell death was more prevalent than neuronal generation during the early phase of retinal development. We further confirmed the relevance of cell death by specific labeling of a subpopulation of nascent RGCs, which were double-labeled for BrdU and Tuj-1 using the window-labeling technique [12]. These results confirmed our previous findings regarding the selectivity of cell death in RGCs [14, 20] and revealed that cell death mediates a 50% reduction in the number of recently generated RGCs shortly after cell birth.

The present findings add to a growing, albeit still overlooked, body of evidence demonstrating the relevant early neural cell death during development. The relative magnitude of this process, as described here during retinal neurogenesis, indicates that it plays a key role in the developmental process, in agreement with the massive apoptosis observed during cortical development in the mouse [21]. Moreover, transgenic mice in which cell death is prevented exhibit dramatic expansion of nervous tissue and, in some cases, embryonic lethality (reviewed in [10]). In addition to experimental data, mathematical models have demonstrated that levels of cell death up to and exceeding 50% are compatible with normal neural development [5].

The results of this study suggest that neuronal cell death is intrinsically linked to RGC generation and neural development. For instance, murine cortical interneurons display similar rates of cell death *in vivo*, in culture, and after transplantation [22]. Further studies are required to determine whether this hypothesis can also be applied to other neural cell types and phases of cell death. Among other possible intrinsic cell death determinants, the generation and repair of DNA double-strand breaks have been consistently associated with neural cell death and may underlie the induction of cell death and the generation of phenotypic diversity. Indeed, we previously reported that in SCID mutant mice, in which DNA repair is compromised, RGCs selectively undergo extensive cell death [23]. Although the existence of somatic mosaicism in the nervous system was proposed over 30 years ago [24], recent studies combining neural stem cells, single cell analysis, and ultrasequencing approaches have allowed for a much more detailed analysis than previously possible. LINE-1 retrotransposition has been implicated in this process, although the results remain controversial [25, 26]. Furthermore, genetically modified mice, in which DNA repair is impaired exhibit massive neural cell death (reviewed in [10, 27]). 

In conclusion, we have established in the developing chick and mouse neuroretinas the pace and relative magnitude of proliferation, differentiation, and cell death. Cell death is preferentially associated with neurogenesis and its relative magnitude exceeds that of neuronal differentiation. Precise quantization of these processes in the embryonic chick retina reveals that half of the recently generated retinal ganglion cells die shortly after birth. Taken together, our results support that cell death is a genuine developmental process and may facilitate future studies of the function of cell death in early neural development.
